# Alterations in the ocular surface and tear film following keratoplasty

**DOI:** 10.1038/s41598-022-16191-6

**Published:** 2022-07-14

**Authors:** Jay J. Meyer, Akilesh Gokul, Michael T. M. Wang, Justin Sung, Jennifer P. Craig

**Affiliations:** Department of Ophthalmology, Faculty of Medical and Health Sciences, New Zealand National Eye Centre, University of Auckland, Private Bag 92019, Auckland, New Zealand

**Keywords:** Corneal diseases, Eye abnormalities

## Abstract

The purpose of this study was to evaluate any alterations in the tear film and ocular surface beyond the early postoperative period following penetrating keratoplasty (PK) and deep anterior lamellar keratoplasty (DALK). This cross-sectional, contralateral-eye study compared ocular surface and tear film parameters of eyes with a previous PK or DALK in one eye and no prior surgery in the contralateral eye. Overall, 14 (87.5%) participants underwent PK, and 2 (12.5%) underwent DALK using a mechanical dissection. The median time from surgery was 3.4 years (range 1.5 to 38.7 years). The indication for unilateral keratoplasty was keratoconus in 15 (94%) participants, and corneal scarring in 1 (6%) eye, secondary to microbial keratitis. Operated eyes exhibited poorer non-invasive tear film breakup time, lower corneal sensitivity, lower sub-basal nerve density and more severe fluorescein staining scores than unoperated fellow eyes (all Q < 0.05). There were no significant differences in tear film lipid layer quality, tear meniscus height, conjunctival hyperaemia, lissamine green staining score, or meibography grade between operated and fellow eyes (all Q ≥ 0.20). Higher corneal esthesiometry threshold (lower corneal sensitivity) was correlated with shorter non-invasive tear film breakup time (Spearman’s rho =  − 0.361, p = 0.04) and increased fluorescein staining score (Spearman’s rho = 0.417, p = 0.02). Keratoplasty can induce persistent changes in the ocular surface and tear film, including: increased fluorescein staining, decreased tear film breakup time, decreased corneal sub-basal nerve plexus density, and reduced corneal sensitivity.

## Introduction

Surgically-induced alterations of the ocular surface are a well-recognised cause of dry eye disease (DED)^1^. Numerous studies have described the findings and pathophysiology of DED following corneal refractive surgery^2–14^ and cataract surgery^15–23^. Dry eye disease is often incited or exacerbated by these surgeries, with a transient increase in symptoms that may last up to 3 months^15–18,21,24^, although a small proportion of patients may have chronic symptoms that persist for over a year^4^. Corneal denervation and decreased corneal sensation have been described as significant causes of postoperative DED following both cataract surgery and refractive surgery^1,24,25^.

In comparison, relatively little is known about the characteristics of DED in eyes that have undergone prior keratoplasty, particularly beyond the immediate postoperative period. A better understanding of the characteristics and mechanisms of DED following keratoplasty is needed, as DED has been identified as a risk factor for complications, including microbial keratitis^26,27^ and corneal graft rejection^28^. Prior studies have examined DED in the first year following keratoplasty, with conflicting results. This cross-sectional study was performed to assess the longer-term alterations in the tear film and ocular surface in eyes with prior, unilateral, penetrating keratoplasty (PK) or deep anterior lamellar keratoplasty (DALK), with the contralateral, unoperated eye serving as the control.

## Materials and methods

This cross-sectional, contralateral-eye comparison adhered to the tenets of the Declaration of Helsinki, and was approved by the University of Auckland Human Participants Ethics Committee (#016663) and informed consent was obtained from all participants. Inclusion criteria required previous PK or DALK in one eye and no prior surgery in the contralateral eye. Exclusion criteria included age < 18 years, transplantation within 1.5 years of examination, the presence of corneal sutures, current topical corticosteroid therapy, contact lens use within the previous 2 weeks, postoperative complications such as infection or rejection, diabetes, and any ocular surgery other than the corneal graft.

The Ocular Surface Disease Index (OSDI), McMonnies, and SPEED questionnaires were administered to grade dry eye symptomology. To minimise the impact on ocular surface and tear film physiology for subsequent tests, measurements were performed in ascending order of invasiveness: blinking assessment, tear meniscus height, tear film lipid layer grade, conjunctival hyperaemia, non-invasive tear film breakup time (NIBUT), slit lamp examination, ocular surface staining, central corneal sensitivity, and confocal microscopy. All participants were assessed in the same room.

Participant blinking was manually observed by the examiner during enrolment while the participant was unaware and not engaged in any visually demanding tasks. The blink rate was calculated as the total number of complete and incomplete blinks in a 60 s period. Tear meniscus height, non-invasive tear film breakup time (NIBUT), tear film lipid layer grade, conjunctival hyperaemia, meibography score and incomplete blinking were assessed with the Oculus Keratograph 5M (Oculus Optikgeräte GmbH, Wetzlar, Germany). The lower tear meniscus height was assessed using high magnification pre-calibrated digital imaging, by taking the average of three measurements near the centre of the lower meniscus. Non-invasive tear film breakup time was measured using automated detection of the first break-up while the subject maintained fixation and was requested to refrain from blinking. When available, three breakup time readings were averaged in each case^29^. Bulbar conjunctival hyperaemia was evaluated by automated objective evaluation of high magnification digital imaging, using the proprietary JENVIS grading scale from 0 to 4^30^. Tear film lipid layer grade was determined with interferometry according to the modified Guillon–Keeler grading system: grade 1, open meshwork; grade 2, closed meshwork; grade 3, wave or flow; grade 4, amorphous; grade 5, colored fringes; grade 0, non-continuous layer (non-visible or abnormal colored fringes)^31,32^.

Fluorescein and lissamine green staining were graded by applying an Oxford grading score, of between 0 and 5, to 6 conjunctival and 5 corneal zones to provide a total score out of 55. Incomplete blinking was deemed present if sclera or cornea remained visible at the point of maximal lid closure and occurred during a period of natural blinking between ocular assessments. Assessment for incomplete blinking was performed using infrared video recording collected by the Oculus Keratograph 5M and also by manual observation during assessment of sodium fluorescein ocular surface staining^33^.

Central corneal sensitivity was determined using non-contact air-jet esthesiometry (NCCA, SDZ Electronics, New Zealand). A barely susceptible flow of air was used to determine the sensitivity threshold via a forced-choice double-staircase method^34^. Sensitivity thresholds were measured in a quiet room devoid of distractions and a standardised 10 mm working distance from the geometric centre of the cornea. Participants were instructed to blink frequently to avoid excessive ocular surface drying and possible dampening of the sensitivity threshold^34,35^.

Evaluation of the corneal sub-basal nerves was performed using the Heidelberg Retina Tomograph III Rostock Corneal module (Heidelberg Engineering, GmBH, Germany) by scanning the full central corneal thickness. Three compression-free images of each of the sub-basal corneal nerve plexus were selected. Quantitative analysis was performed using the Image J software (U. S. National Institutes of Health, Bethesda, Maryland, USA, available in the public domain at http://rsb.info.nih.gov/ij/). The Neuron J plugin for Image J was utilised to produce semi-automated calibrated (394 pixels = 400 µm) tracings of the sub-basal nerve plexus. All nerves contained within the entire 400 × 400 µm image were traced and subsequently converted to a nerve density. All images were anonymised and randomised prior to analysis. The mean of the quantitative analysis of three images was utilised to obtain the sub-basal corneal nerve density (mm/mm^2^).

The sample size was pragmatically determined by the number of participants meeting the enrolment criteria during the study period. Normally distributed continuous variables were compared with a paired t-test, and non-normal and ordinal data, with the Wilcoxon signed-rank test. False discovery rate adjustment of p-values was applied and reported as Q-values to account for multiple comparisons. Secondary analyses assessing the association between corneal esthesiometry threshold and other ocular surface parameters as well as blink rate were performed using Spearman's rank correlation coefficient. All tests were two-tailed and Q < 0.05 was considered significant.

## Results

The mean ± SD age of the 16 participants (9 males, 7 females) was 38 ± 14 years. Overall, 14 (87.5%) participants underwent PK, and 2 (12.5%) underwent DALK using a mechanical dissection. The median time from surgery was 3.4 years (range, 1.5 to 38.7 years). The indication for unilateral keratoplasty was keratoconus in 15 (94%) patients, and corneal scarring in 1 (6%) participant, secondary to microbial keratitis. The mean ± SD OSDI dry eye symptomology score was 38 ± 23, McMonnies score was 11 ± 6, and SPEED score was 11 ± 7. The mean blink rate was 13 ± 5 per minute, and incomplete blinking was observed in 3 (19%) of subjects.

Measurements from the post-keratoplasty eyes and unoperated fellow eyes are summarised in Table 1. Operated eyes exhibited poorer non-invasive tear film breakup time, higher corneal esthesiometry threshold (lower corneal sensitivity), lower sub-basal nerve density and more severe fluorescein staining scores than unoperated fellow eyes (all Q < 0.05). There were no significant differences in tear film lipid layer quality, tear meniscus height, conjunctival hyperaemia, lissamine green staining score, or meibography grade between operated and fellow eyes (all Q ≥ 0.20).

**Table 1:** Measurements of post-keratoplasty eyes and unoperated fellow eyes.

Parameter	Keratoplasty eye (n = 16)	Fellow eye (n = 16)	p-value	Q-value
**Tear film quality**				
Non-invasive tear film breakup time (s)	5.1 ± 3.6	7.6 ± 4.5	0.02*	0.04*
Tear film lipid layer grade (out of 5)	2 (2–3)	3 (2–3)	0.56	0.73
Tear meniscus height (mm)	0.38 ± 0.13	0.40 ± 0.14	0.68	0.73
**Ocular surface characteristics**				
Bulbar conjunctival hyperaemia (out of 4)	1.3 ± 0.7	1.2 ± 0.5	0.28	0.50
Limbal conjunctival hyperaemia (out of 4)	1.5 ± 0.6	1.4 ± 0.4	0.12	0.27
Corneal esthesiometry threshold	0.6 (0.3–1.0)	0.4 (0.2–0.8)	< 0.001*	< 0.001*
Sodium fluorescein staining score (out of 55)	4 (0–8)	2 (0–5)	0.004*	0.02*
Lissamine green staining score (out of 55)	2 (0–4)	2 (0–4)	0.91	0.91
Superior meibography grade (out of 4)	1 (1–3)	1 (1–3)	0.73	0.73
Inferior meibography grade (out of 4)	0 (0–1)	0 (0–1)	0.62	0.73
**In vivo confocal microscopy**				
Sub-basal nerve density (mm/mm^2^)	6.30 ± 6.40	20.6 ± 9.2	< 0.001*	< 0.001*

Data are presented as mean ± SD, median (IQR), or number of participants (% of participants).

Asterisks denote statistically significant differences (Q < 0.05).

Secondary analyses assessing the association between ocular surface surface parameters demonstrated that higher corneal esthesiometry threshold (lower corneal sensitivity) was correlated with shorter NIBUT (Spearman’s rho =  − 0.361, p = 0.04) and increased sodium fluorescein staining score (Spearman’s rho = 0.417, p = 0.02). No significant correlations were detected between corneal esthesiometry and other ocular surface parameters (all p > 0.10). No significant correlations were detected between the blink rate and corneal esthesiometry of operated (Spearman’s rho =  − 0.241, p = 0.37) or control eyes (Spearman’s rho =  − 0.358, p = 0.17).

## Discussion

In this study, post-keratoplasty findings were compared to those of the contralateral eye, to evaluate the longer-term impact of keratoplasty on the ocular surface and tear film. A few retrospective and prospective studies have examined the tear film characteristics within the first year following PK and DALK, with variable and conflicting results. Some studies have reported decreased tear film stability^36,37^ while others have reported increased stability^38,39^. Similarly, no changes in tear volume^36,38^ as well as an increase in tear volume^37^ have both been reported following keratoplasty. One study of eyes following PK reported improved OSDI scores and corneal staining postoperatively, despite decreased Meibomian gland expressibility and quality^39^. Incongruence in the outcomes of these studies may reflect differences in the patient ages, graft characteristics, underlying diagnoses, and study methodologies. In addition, the evaluation of DED within the first year following keratoplasty is confounded by many possible factors, including corneal sutures, irregularities of the graft-host junction, postoperative medications such as topical corticosteroids and preserved lubricants, and inflammation. For these reasons, we sought to investigate the ocular surface characteristics of eyes beyond this early postoperative period.

We identified increased fluorescein staining, decreased NIBUT, decreased sub-basal nerve plexus density, and reduced corneal sensitivity in eyes with prior keratoplasty compared to the contralateral, control eyes. All other measured ocular surface and meibomian gland parameters were not statistically different between the groups of eyes. Similar findings have been identified after cataract and refractive surgery, with a decrease in TBUT and increase in corneal staining identified postoperatively, along with variable changes in the tear volume^15–18,21,22^.

Corneal transection or ablation and a decrease in corneal sensation have been described as causes of postoperative dry eye following both cataract and corneal refractive surgeries^1,24,25^. The degree of corneal denervation following refractive surgery may vary depending on the type of surgery, ablation depth, mechanism of flap creation, and possibly flap hinge location^4,5,40,41^. Corneal incision characteristics at the time of cataract surgery, such as the size (width, length) and architecture (grooved vs. planar) may also influence the degree of corneal transection^16,18^. Regeneration of corneal nerves occurs over approximately 3 months following cataract surgery and 3 to 6 months after laser in situ keratomileusis (LASIK), although innervation may not reach preoperative levels even at 1 year following LASIK^16,42^. Corneal sensitivity is decreased for approximately one month following phacoemulsification and 3 months following LASIK^42^. Dry eye symptoms generally subside over a similar time course.

Compared to cataract and refractive surgery, the corneal denervation following PK and DALK is much more profound and persistent. PK and DALK require a 360° full-thickness (PK) or near full-thickness (DALK) corneal incision that transects the corneal nerves and results in total denervation of the transplanted cornea. While there is partial regeneration of corneal nerves with time, this reduction and alteration of the sub-basal plexus can persist for over 40 years^43^. A decrease in corneal sensitivity within the first 12 months following PK or DALK has previously been reported^37,38^. In the present study, it was identified that reduced corneal sensitivity can persist beyond the first year following transplantation. Such reduced sensitivity, likely a result of reduced sub-basal innervation, may interfere with the neural feedback loop of tear production, although the relationship between DED and corneal sensitivity is not well defined^36,38^.

It has been proposed that following LASIK, reduced corneal sensitivity may reduce the blink rate and subsequently destabilize the tear film through increased evaporation^24^. No correlation between blink rate and corneal sensitivity was identified in the present study, although it was not designed to specifically assess this association. In addition, any association between decreased corneal sensitivity and blink rate might be expected to be more significant following bilateral decreased corneal sensitivity such as bilateral LASIK or other corneal surgeries, while we only studied eyes with unilateral surgery. Decreased meibum secretion secondary to inadequate blinking has also been hypothesized^24,44^, and it is unknown whether corneal denervation could also affect meibomian glands through disruption of their neural regulation^39^. Neural disruption of the cornea might also affect expression of membrane-associated mucins on the cornea, leading to decreased tear stability^24,45^.

Other potential surgery-related mechanisms of DED have been proposed, including the use of topical anesthetics, exposure desiccation, microscope light exposure, incitement of inflammatory mediators, Meibomian gland dysfunction, and goblet cell loss^16–18,21,25^. Following keratoplasty, the irregular corneal surface could also affect tear film distribution and stability.

There are several limitations of this study. Only those patients without current contact lens wear were studied, in order to reduce any confounding effects of the contact lens wear on the study results. However, this limited the sample size and associated statistical power of the study and could have potentially resulted in a study population more likely to have had prior contact lens intolerance due to dry eye symptoms or discomfort. Also, the cross-sectional nature of the study does not exclude the possibility that differences in the studied parameters between contralateral eyes could have been present preoperatively and also did not allow the determination of the magnitude of any changes in the parameters over time. Almost all the eyes in this study had bilateral keratoconus, which in itself can alter corneal innervation through reduced density and abnormal morphology of the sub-basal nerve plexus and decreased keratocyte density^46^. The contralateral-eye comparison nature of the study design sought to minimise the influence of such co-morbidities on the outcomes, but the possibility cannot be excluded. In addition, we did not perform evaluation of the graft-host junction surface profile which could influence the tear film, particularly its stability, due to corneal surface irregularities. Thus, it cannot be confirmed whether the reduced NIBUT in eyes with prior keratoplasty was secondary to corneal surface irregularities at the graft host junction affecting the distribution of the tear film, due to alterations in the tear film itself, or due to a combination of these mechanisms.

In conclusion, keratoplasty can induce persistent changes in the ocular surface and tear film that contribute to signs and symptoms of DED. Further research is required to elucidate the exact mechanisms of some of these findings and determine whether any specific interventional strategies are beneficial.

## Data Availability

The datasets generated and analysed during the current study are not publicly available as public data sharing was not included in the ethics committee approval.
